# GBS vaccines in the UK: a round table discussion

**DOI:** 10.12688/f1000research.147555.1

**Published:** 2024-05-21

**Authors:** Natasha Thorn, Rebecca L Guy, Konstantinos Karampatsas, Mair Powell, Kate F Walker, Jane Plumb, Asma Khalil, Vanessa Greening, Emma Eccleston, Caroline Trotter, Nick Andrews, Lynne Rush, Claire Sharkey, Lauren Wallis, Paul Heath, Kirsty Le Doare

**Affiliations:** 1 St George's University of London, London, SW17 0RE, UK; 2 UK Health Security Agency, London, UK; 3 Healthcare Products Regulatory Agency, Dublin, Ireland; 4 University of Nottingham, Nottingham, England, UK; 5 Group B Strep Support (GBSS), Haywards Heath, UK; 6 Imperial College London, London, England, UK; 7 University of Cambridge, Cambridge, England, UK; 8 Public Health Scotland, Edinburgh, UK

**Keywords:** Group B streptococcus, invasive GBS disease, vaccines, maternal vaccines, late-onset disease, early-onset disease, neonate

## Abstract

**Background:**

Group B streptococcus (GBS) remains a leading cause of infant sepsis, meningitis and death despite intrapartum antibiotic prophylaxis. A vaccine is urgently required, and two candidates are in advanced clinical trials. For successful GBS vaccine implementation, especially if a vaccine is licensed based on an immunological threshold, there must be cross-sector engagement, effective advocacy, robust plans for phase IV studies and equitable access.

**Meeting:**

A round-table discussion, held at St George’s University of London, reviewed the current position of GBS vaccines in the UK context, focusing on phase IV plans, convening a diverse group of stakeholders from across the UK, with a role in GBS vaccine licensure, advocacy, implementation or effectiveness evaluation.

Presentations outlined the latest UK epidemiology, noting the rising infant invasive GBS (iGBS) infection rates from 1996 to 2021 for both early and late onset disease, with the highest disease rates in Black infants (1.1/1000 livebirths vs white infants (0.81/1000 livebirths). Potential coverage of the candidate vaccines was high (>95%). Regulatory input suggested that EU regulators would consider waiving the need for a pre-licensure efficacy study if a putative correlate of protection could be adequately justified. Phase IV study methodologies for a GBS vaccine were considered, largely based on previous UK maternal vaccine assessments, such as a nationwide cohort study design using a vaccine register and a maternal services dataset. Other strategies were also discussed such as a cluster or stepped-wedge randomised trial to evaluate implementation outcomes. Opportunities for advocacy, education and engagement with additional key partners were discussed and identified.

**Conclusions:**

With an approved GBS vaccine a near possibility, planning of phase IV studies and identification of critical barriers to implementation are urgently needed. Cross-sector engagement is essential and will facilitate a successful pathway.

## Introduction and Objectives

*Streptococcus agalactiae* or group B *streptococcus* (GBS) remains an important cause of neonatal sepsis and meningitis worldwide. ^1^^–^^4^ It represents a high global burden of infant morbidity and mortality, ^2^ causing a spectrum of disease including neonatal sepsis, pneumonia, meningitis and long-term neurological sequelae in survivors. ^2^^,^^5^^–^^7^ In pregnancy, GBS causes bacteriuria, ascending infection and chorioamnionitis as well as maternal perinatal sepsis and death. ^2^^,^^5^^,^^8^ It is associated with excess risk of preterm birth and stillbirth. ^2^^,^^9^^–^^12^ In 2020, it was estimated that globally, GBS caused 394,000 cases of infant invasive GBS (iGBS) disease and 91,900 infant deaths as well as being associated with 518,000 excess preterm births. ^1^

Whilst the heaviest burden of GBS falls on low- and middle-income countries (LMICs), there remains significant disease in the UK with recent, concerning trends. Decreased rates of early-onset GBS (EOGBS) disease, occurring within 0 to 6 days of life, have been seen in most high-income countries after the introduction of intrapartum antibiotic prophylaxis (IAP), especially where a test-based rather than risk-based approach is taken. However, this intervention has not reduced rates of late-onset GBS (LOGBS) disease, occurring between 7 to 90 days of life. ^13^ In some high income countries early-onset disease (EOD) rates have been rising in recent years, including in the UK ^14^ where a risk-based strategy for IAP is used. IAP is not always deliverable, results in high antibiotic exposure leading to concerns about increasing antimicrobial resistance (AMR) ^13^^,^^15^^,^^16^ and has unwanted effects on the neonatal microbiome in the short-term. ^17^ With no effective prevention strategy for LOGBS and rising rates of EOGBS, a GBS vaccine remains a significant, long-standing unmet need in the UK.

Vaccines are in development and two vaccine candidates have reached phase II trials in pregnant people; a hexavalent, capsular polysaccharide-protein conjugate vaccine ^18^ and an Alpha-like protein (Alp) adjuvanted vaccine. ^19^ An effective GBS vaccine has the potential to prevent an estimated 127,000 (UR: 63,300 to 248,000) EOGBS cases and 87,300 (UR: 38,100 to 209, 000) LOGBS cases globally every year. ^20^

However, phase III trials present significant challenges ^21^; primarily that they will require a very large sample size. In the UK, where invasive disease incidence is below 1 per 1000 livebirths, over 150,000 pregnant participants would be required to demonstrate 75% vaccine efficacy. ^22^ Alternative pathways to licensure are therefore being pursued with establishment of an immune correlate of protection (COP) one option. ^23^^–^^25^ Modelling in the UK has demonstrated a vaccine likely to be cost effective at both £20,000 and £30,000 per quality-adjusted life year (QALY). ^26^ Cost-effectiveness is also predicted across multiple settings. ^20^

The UK has a history of vaccine innovation and early adoption of new vaccines. So with GBS vaccines on the horizon there are a multitude of considerations for stakeholders in the UK to ensure a rapid and accessible vaccine programme with good uptake. With this in mind, a round table discussion was facilitated, gathering an expert group of stakeholders from across the UK.

The meeting, held in March 2023 at St George’s University, London (SGUL), included presentations and group discussion. Future meetings will focus on areas requiring greater depth and to progress next steps. Here, we report on the first meeting, including proposed next steps along the road to a GBS vaccine programme for the UK.

### Epidemiology and Surveillance

***Burden of invasive GBS disease in the UK – Rebecca Guy***

The first presentation was given by Rebecca Guy, principal scientist in epidemiology at the UK Health Security Agency (UKHSA) outlining the current burden of invasive GBS (iGBS) disease in the UK. Several data sources were used, including the UKHSA routine lab surveillance data of GBS isolated from sterile sites in England (SGSS), data from the Staphylococcal and Streptococcal Reference Service (SSRS), information from the NHS demographic batch services (DBS) from the NHS Spine and hospital episode statistics (HES). Some of the data presented were unpublished.

iGBS disease incidence trends in England from 2017 to 2021 remain broadly stable across this period at around 5 per 100,000 population in all ages. Incidence trends consistently differ between age groups; the highest incidence rate is in under 1-year olds at 90/100,000 population, followed by the over 75-year-olds at 14/100,000 population.

Infant iGBS infection rates have increased markedly between 1996 to 2021, for both EO and LO disease. Infant disease rates reached 0.95/1000 livebirths in 2019. There was a 40% increase in EOD between 2012 and 2020 (0.37 and 0.53/1000 livebirths respectively). During the two periods of enhanced surveillance in the UK and Ireland in 2000/01 and 2014/15, there was a comparable increase in incidence, suggesting slight under-ascertainment by routine surveillance data.

In infant iGBS from 2010 to 2021, capsular serotypes from England, Wales and Northern Ireland (NI) continue to differ between EOD and LOD. EOD demonstrates more serotype diversity, with type III still predominant but declining alongside decreased serotype Ia and increased IV in recent years. Looking cumulatively at infant iGBS isolate serotypes from sterile sites 2017 to 2021, 73% of LOD are due to serotype III but just 42% of EOD. 95% of infant disease isolates from 2017 to 2021 would be covered by the hexavalent vaccine currently in clinical trials. It is noted that this data represents the subset of cases referred to SSRS. Improvements to microbiology surveillance would be beneficial prior to a vaccine being launched.

Antimicrobial resistance (AMR) is increasing in England in iGBS cases. In 2021, 40% of isolates from invasive disease in under 1-year olds had erythromycin, and 36% clindamycin resistance. Despite the change in IAP guidance in 2017 reducing use of clindamycin, this trend has continued. Improved surveillance is important ahead of vaccine introduction to assess whether trends are of emerging resistance or expansion of serotypes.

Reporting on infant iGBS infections by ethnicity in England, 2019 to 2020, the highest disease rates are in Black infants (1.1/1000 livebirths) and lower in white (0.81/1000 livebirths) and mixed ethnicity infants (0.6/1000 livebirths). The infection rate in Black infants is therefore 36% higher than in white infants. There is also significant variability between Asian infant subgroups. The same findings apply to maternal ethnic variation in iGBS disease; iGBS is highest overall in the mothers of Black infants (0.69/1000 livebirths) at more than twice the rate of white mothers (0.29/1000 livebirths). iGBS in England by deprivation decile from 2015 to 2021, for all ages, demonstrates a consistent trend indicating cases are reported more frequently from deprived areas. One previous study with UKHSA at a London hospital showed differentials in carriage rates between ethnicities ^27^ which may provide part of the explanation but further work is needed to understand the whole picture and is being undertaken in a partnership between University of Nottingham and UKHSA.

The group discussed UKHSA’s genomic work on GBS looking at potential linkages between cases. Although LOGBS has traditionally been thought of as individually acquired community cases, the genomic work showed that 1 in 12 isolates were linked in hospital clusters and so it can also be thought of as a hospital acquired infection (HAI).

***The Scottish perspective on current GBS surveillance – Lynne Rush***

Lynne Rush gave the Scottish perspective. There is not currently enhanced disease surveillance for GBS in Scotland. Isolates are received via the Scottish microbiology reference laboratories, but these are not linked to clinical data. This would need to be addressed in a systematic way, preferably in line with the rest of the UK. Public Health Scotland (PHS) have good connections with the MatNeo Data Hub in partnership with the Scottish Perinatal Network, Healthcare Improvement Scotland, Scottish Government and National Records Scotland which will be a valuable framework to work with going forwards.

In terms of vaccine coverage data, historically antenatal vaccine uptake has been challenging to record but Scotland has recently been through the Vaccine Transformation Programme leading to major changes including use of the new Scottish Linked Pregnancy and Baby Dataset (SLiPBD), the new Vaccine Management Tool and changes to how vaccines in pregnancy are captured.

PHS has a robust system for monitoring vaccine safety, with weekly adverse events reported from the NHS board of immunisation co-ordinators discussed weekly at a vaccine safety team meeting. In terms of cost-effectiveness studies, the Scottish birth cohort is approximately 50,000 – a sample size too low to power a stand-alone study for Scotland and therefore being part of a UK-wide trial would be welcomed.

***Proposed surveillance of iGBS disease in British and Irish infants <90 days – Konstantinos Karampatsas***

A presentation by Konstantinos Karampatsas, clinical research fellow at SGUL, introduced a submitted proposal for a new enhanced surveillance study looking at iGBS disease in infants < 3 months of age in the UK and Republic of Ireland. The most recent enhanced surveillance for GBS in the UK was the British Paediatric Surveillance Unit (BPSU) study in 2014-15. ^14^ 856 infants were identified over 13 months giving an incidence of 0.94 cases/1000 livebirths and 53 infants died meaning a case fatality rate (CFR) of 6.2%. Serotype III was the most prominent serotype in both EOD and LOD (60% of cases) followed by serotype Ia (17% of cases). One of the most important conclusions from this study was that the incidence of GBS had significantly increased from the previous BPSU study in 2000 for both EOD and LOD, which means that the burden of EOGBS has increased despite the national guidelines introducing a risk-based policy for IAP.

The need for a new surveillance study was highlighted: it will provide baseline information ahead of both a test-based screening and vaccine programme to accurately assess their impact. It will describe the current prevalence of clinical risk factors e.g., prematurity; and will help to identify whether at-risk groups will be covered by a vaccine during pregnancy. The study will also describe any changes in the incidence of iGBS and assess the effect of the implementation of the current IAP guidelines. Additionally, it will help describe serotypes circulating to bolster confidence that the vaccines in development will offer adequate cover.

The proposed study design uses the BPSU network in collaboration with microbiology reference laboratories and public health agencies in the UK and Ireland. The team will also collaborate with the UK Obstetric Surveillance System (UKOSS), raising awareness amongst obstetricians and midwives and helping to capture critical data around pregnancy and birth. The team are aiming to consult with PPI organisations to promote transparency and accountability as a non-consent model will be used.

The definition of cases at present is GBS identified via positive culture or polymerase-chain reaction (PCR) test from a normally sterile site. Engaging with hospital laboratories will be key to the success of this project, especially given that only 45% of all isolates are currently sent to the reference laboratories in the UK. The BPSU also recommended an additional timepoint in the study with a follow up at 2 years of age to assess long-term morbidity of iGBS.

A Phase 1 application was accepted in December 2022 and the Phase 2 application is being updated before approval, with a plan to meet shortly with all laboratories and agencies to discuss logistics. The study will commence within 12 months of phase 2 approval.

### Epidemiology and Surveillance: Knowledge Gaps Identified and Next Steps

Group discussion identified epidemiology and surveillance knowledge gaps in the context of a GBS vaccine programme for the UK. Table 1 details these knowledge gaps as well as next steps to begin to address these.

**Table 1.** Epidemiology and surveillance: knowledge gaps identified and next steps.

Knowledge gaps identified	Next steps
*Stillbirth:* GBS-associated stillbirth is felt to be underestimated due to lack of standardised assessment of aetiology for both intra and antepartum events. Stillbirth due to GBS is only included in UK EOGBS data if there has been a microbiological diagnosis made, which is by no means universal. Clearer understanding of GBS associated stillbirth in the UK will allow a clearer picture of GBS burden ahead of a vaccine and may help contribute to cost-effectiveness analysis.	*Identify potential data sources:* The GBS3 trial team will be able to provide some data around this in the UK as they are adjudicating all stillbirths within the trial. A proposed package of work to address GBS related stillbirths via a collaboration between SGUL, University of Oxford, the national Mothers and Babies: Reducing Risk through Audits and Confidential Enquiries collaboration (MBRRACE-UK), GBSS and perinatal pathologists. A vaccine probe study could contribute data here
*Incidence disparities:* Variation in iGBS incidence rates between ethnicities and at differing deprivation levels is an important standalone issue. In relation to a GBS vaccine, high deprivation level and ethnic minority status are both risk factors for poor vaccine uptake in pregnancy. ^28^^,^^29^ It is unclear what all the contributing factors to these disparities are.	To ensure equitable access to a GBS vaccine, deprivation and ethnicity in relation to iGBS disease incidence should be further explored and understood in greater depth. *Potential data sources for this:* National level incidence, ethnicity and deprivation data from UKHSA – a more detailed exploration GBS3 trial dataset at completion The proposed enhanced surveillance study data The group will consider this again at further meetings.
*Microbiological surveillance data:* This could be strengthened ahead of vaccine implementation. Increased rates of isolate referral to reference laboratories would improve assessment of resistance patterns and circulating serotypes ahead of a vaccine programme.	*Considerations suggested by the group were:* GBS could be made a mandatory reporting organism at national laboratory level - whether this is proportionate will be discussed. Given some LOGBS cases are linked in hospital clusters, GBS could be included in routine nosocomial pathogen surveillance to increase understanding of transmission routes. The proposed enhanced surveillance study should improve isolate referrals during that period. Whole genome sequencing of GBS infant disease isolates would be helpful as part of the proposed surveillance study. Main barrier at present is availability of funding.
*Neurodevelopmental outcomes:* There is a lack of data around longer-term neurodevelopmental (ND) outcomes in infant GBS survivors in the UK.	In addition to the proposed enhanced surveillance study via the BPSU, the SGUL team will undertake a ND follow-up study at 2 years of age. This is being further developed, defined and consulted on to ensure this opportunity is maximised.

### Progress Towards a GBS Vaccine in Pregnancy

***GBS Vaccines – Paul Heath***

A presentation from Paul Heath, Professor of Paediatric Infectious Diseases at SGUL provided an update on GBS vaccine development. An effective GBS vaccine could prevent an estimated global 127,000 EOGBS cases (UR: 63,300 to 248,000), 87,300 LOGBS cases (UR: 38,100 to 209, 000) averting 31,100 infant deaths (UR: 14,400 to 66, 400) and 17,900 cases of moderate and severe neurodisability (UR 6,380 to 49,900). ^20^

There are two main GBS vaccine targets – the capsule and it’s 10 different serotypes, and several conserved outer membrane proteins targets. The global GBS capsular serotype distribution is important because one of two current leading vaccine candidates is a serotype-specific vaccine, covering the six most frequent serotypes. Serotype replacement is therefore a theoretical possibility, as has been seen with pneumococcal conjugate vaccines, although perhaps less likely for GBS.

To date, vaccine candidates have been developed and trialled by multiple companies but the two most advanced currently are by Pfizer and MinervaX. The Pfizer vaccine is a six-valent polysaccharide protein conjugate vaccine ^18^ whilst MinervaX have an Alpha-like protein (Alp) based vaccine. ^19^ Both manufacturers are in phase II trials in pregnant people and are currently planning phase III trials, grappling with the issues around route to licensure: primarily, large sample size requirement.

A 2021 study of the polysaccharide conjugate vaccine demonstrated immunogenicity and persistence against 6 serotypes in non-pregnant adults as part of a dose-finding study. ^30^ This study ultimately chose a 20mcg per serotype dose, finding the aluminium phosphate adjuvant not necessary for immunogenicity. The protein vaccine is based on the Alp proteins which are conserved across nearly all strains and is being tested in pregnant participants. It uses an aluminium hydroxide adjuvant.

Some of the issues to consider ahead of a vaccine programme are: reactogenicity and immunogenicity of co-administered vaccines in pregnancy (for example dTaP/IPV, COVID-19, RSV, influenza and tetanus), optimal timing of vaccine(s) in pregnancy as placental transfer is maximal in the 3 ^rd^ trimester yet premature infants experience the highest incidence of GBS, attitudes towards antenatal vaccination. Other issues include impact on colonisation and the persistence of antibodies in infants and also in adults into future pregnancies: will this vaccine be given in every pregnancy? Finally, any interference with primary immunisations must be evaluated, given that pertussis and polio vaccines in pregnancy can affect infant responses. ^31^

***Serocorrelates as the basis for conditional licensure of vaccines in the UK – Kirsty Le Doare***

A presentation from Kirsty Le Doare, Professor of Vaccinology and Immunology at SGUL, gave an overview of the serocorrelates route to GBS vaccine licensure. There are three potential approaches to vaccine licensure currently; the first, the classic approach, is a phase III trial. The second approach is an immunological endpoints trial embedded within a classic phase III trial. The third option is conditional approval based on an immunological endpoint followed by a phase IV clinical effectiveness trial. At present, the second two options are the most likely routes for a GBS vaccine. To demonstrate efficacy, a larger sample size is needed than would generally be practical for a phase III trial in pregnancy. ^22^ Even in a high disease burden country, with a disease incidence of 1.0-2.0/1000 livebirths, for a 1:1 randomised, controlled GBS clinical vaccine efficacy trial, 30-62,000 participants would need to be enrolled to detect vaccine efficacy of 80%. ^23^

A basis for licensure based on serocorrelates has been used previously for pneumococcal vaccines where an aggregate immunoglobulin G (IgG) serocorrelate value (the amount of antibody that protects an infant from invasive disease) that covered all serotypes was developed based on three clinical efficacy trials and agreed by consensus to be 0.35 μg/ml. ^32^ Importantly, the way forward for licensure was having a standardised assay. A similar approach was taken for meningococcal vaccines where serocorrelates moved the field forward. The premise behind the correlate of protection (COP) is that the birthing-parent has antibodies to GBS post-vaccination or post-exposure to GBS. IgG is passed via the placenta to the fetus and if passed in sufficient amounts, the birthing-parent and baby are protected against disease in either pregnant adult, fetus or infant.

Carol Baker has worked extensively on GBS COP. Baker has spent much time on potential IgG threshold values; her studies from the early 1990’s looked at a tetanus toxoid conjugate vaccine against two of the common GBS serotypes in the USA, serotype Ia and Ib. ^33^ These studies found that the percentage of people with IgG >1 μg /ml, when examining vaccine versus placebo, was much higher in those who were vaccinated with >90% of non-pregnant adults achieving a titre of >1 μg /ml after vaccination. Studies in the UK examining antibody levels post-natural infection found that infants with antibody concentrations of >2 μg/ml were more likely to be protected against iGBS disease when compared to infants with lower antibody concentrations. ^34^ These results have been replicated in a seroepidemiological study from Europe which examined colonised versus non-colonised women and found that those colonised had higher antibody concentrations for all the major serotypes. ^35^ There have subsequently been several modelling studies in recent years including further work from Baker ^36^ which took a Bayesian approach to predict how much antibody is sufficient, with the overall combined estimate that antibody concentrations >1 μg /ml had an estimated 90% protection against serotypes Ia and III, and 70% protection against serotype IV. COP have also been examined and proposed for the AlpN antibodies; relevant for the protein-based vaccine that is now in Phase II trials. These studies predict an estimated 90% reduction in disease with concentrations of 0.4μg/ml of Rib-N IgG and Alp1-N IgG concentrations of 0.11 μg/ml. ^37^

One factor advancing this area more recently has been the development of the international GBS assay standardisation consortium, which SGUL leads. The three objectives of the consortium are:

1. Development of standard reagents and identification and review of assays for standardisation
2. Standardisation of protocols for existing ELISA and functional GBS assays using standard reagents
3. Validation of standard protocols and standard reagents across laboratories to establish a prediction of disease protection.

A 2021 International Symposium on *Streptococcus agalactiae* Disease (ISSAD) report presented data showing good assay correlation between all laboratories which has now enabled larger studies to go forward. For example, the iGBS3 study in the UK, a study in the USA examining 150 dried blood cards with 3:1 controls from prospective surveillance in newborns performed via the CDC, a large trial under way in South Africa and Uganda and additional work via SGUL (the EDCTP PREPARE study) collecting isolates from around Europe and African countries. All these studies will contribute to defining the COP that can be used for conditional licensure.

***Potential cost effectiveness of GBS vaccines – what more do we need to know? – Professor Caroline Trotter***

Professor Caroline Trotter, Epidemiologist and Health Economist at the University of Cambridge and Imperial College London presented on the cost-effectiveness of a GBS vaccine in the UK. A cost-effectiveness analysis modelling study was performed in 2018 ^26^ which concluded that a GBS vaccine in pregnancy would be cost effective, even at a relatively high estimated threshold price. The model estimated cost-effectiveness at £54 per maternal GBS vaccine dose at £20,000/QALY or £71 per dose at £30,000/QALY. There were no barriers shown to cost-effectiveness in this study.

Data from this and other previous studies ^20^^,^^26^ give a strong starting point for the Joint Committee on Vaccination and Immunisation (JCVI) to make robust recommendations to the Department of Health (DOH). A significant difference in cost-effectiveness since 2018 is unlikely. Updating the working economic model for the UK with new epidemiological, surveillance or cost study data will be helpful. Inputs to update include: infant disease incidence and CFR, expected rates of sequelae, costs of acute and long-term care, litigation costs, vaccine details as known and other important outcomes such as stillbirth, preterm birth reduction and maternal sepsis and death prevention.

An element which has changed since 2018 is a more crowded pregnancy vaccine space leading to increased competition. In the previous paper, ^26^ inclusion of maternal deaths and in particular stillbirths, increased the vaccine threshold price. These were not included in the base case as maternal deaths are very uncommon and stillbirths are not technically included within the National Institute for Health and Care Excellence (NICE) framework for cost-effectiveness analysis. Stillbirth was also demonstrated as an important factor in the global cost-effectiveness analysis. ^20^ The economic handling of stillbirths may therefore be an avenue for further research; the UK could put forward both cost-effectiveness arguments for comparison, with and without value of life years lost to stillbirths, using standard life expectancy. Although relatively small in number, inclusion of intrapartum GBS related stillbirth in the model would likely impact the overall cost-effectiveness.

***GBS vaccines for maternal immunisation to protect against invasive disease in early infancy: Regulatory clinical considerations – Mair Powell***

A presentation was given by Mair Powell, Senior Clinical Assessor at the Healthcare Products Regulatory Authority (HRA) in Ireland, chair of the European Medicines Agency (EMA) Vaccine Working Party, member of the EMA Scientific Advice Working Party and member of the EMA Infectious Diseases Working Party.

The factors affecting time to first licensure for a vaccine were outlined: firstly, what will be the basis for demonstrating or inferring efficacy. Pre-licensure efficacy trials may not be needed if: 1. there is an agreed immune COP, or an agreed threshold value to enable prediction of protection from an immune response, or 2. an efficacious, licensed vaccine already exists, with a prior efficacy/effectiveness trial, and the new candidate can be shown to elicit similar immune responses to allow immunobridging. If neither of the above scenarios exist, an efficacy trial will be required if this is feasible.

If an efficacy trial is not feasible, perhaps in the case of a rare, sporadic, episodic or small outbreak of disease then several approaches can be considered. These include: deriving a threshold level of immune response for protection, from a non-clinical model that can be applied to humans, or deriving a threshold value from natural protection, using serological data and disease surveillance. Lastly, human challenge studies can be considered, where appropriate.

Another consideration is the size of the pre-licensure safety database; generally, for a new vaccine, this will be in excess of 3000 persons exposed to the final vaccine formulation and dose regimen. However, this number is negotiable taking into consideration factors such as the vaccine content and platform. Risk management plans and periodic safety update reports are now mandatory in the UK, EU and US. Specific safety issues may need to be addressed either via a large, pre-licensure safety database, and/or a post authorisation safety surveillance (PASS) study.

Considering effectiveness studies, it is recognised that these are unlikely to be conducted by the marketing authorisation holders (MAH) themselves. It is requested that the MAH engages with relevant public health bodies, working with them to develop a plan for a vaccine effectiveness study.

In summary, the data requirement for vaccine licensure and post-licensure is determined on a case-by-case basis. EU regulators have agreed that a pre-licensure efficacy study for a GBS vaccine may be waived if a sponsor, academic body, consortium or otherwise can propose a putative COP or threshold value. Different approaches to identifying putative COPs or threshold values are being taken to date by sponsors, considering both EOD and LOD as well as individual serotypes.

***Summary of current GBS vaccine pre-licensure considerations – Paul Heath***

If a putative COP can be established to allow inference of vaccine protection, this might suffice for initial approval followed by a post-approval vaccine effectiveness study. Such a study might include secondary endpoints in addition to a primary endpoint based on invasive infant disease.

A pre-licensure efficacy study is probably not achievable as a conventional disease-endpoint, phase III, placebo-controlled RCT. This is because relatively low disease incidence mandates a large sample size to evidence efficacy, participant recruitment during pregnancy means follow up of at least 6-12 months with an 8-12 month enrolment period and at least 200 study sites would likely be required. This study would take several years and significant investment to complete, resulting in a delay to vaccine availability. It is possible that one of the two vaccine manufacturers might pursue a large phase III efficacy trial as this remains the gold standard, but for the other, the approach would likely be a conditional licensure approach.

A conditional licensure approach, based on a putative COP, will still require large numbers of pregnant participants, but is less costly than a pre-licensure, phase III clinical trial. Examples of vaccines approved via a COP route include the Meningococcal C, Meningococcal B and Meningococcal ACWY vaccine.

### Progress Towards a Vaccine: Knowledge Gaps Identified and Next Steps

Group discussion identified further knowledge gaps in the area of progression towards a GBS vaccine programme in the UK. Table 2 details these knowledge gaps as well as next steps to begin to address these.

**Table 2.** Progress towards a vaccine: knowledge gaps identified and next steps.

Knowledge gaps identified	Next steps
*Pathway to licensure:* Data is required to provide a COP for a GBS vaccine for the UK population.	There is ongoing work to define a COP for the UK population - SGUL are running an ongoing case control study in relation to this. Collaboration with MHRA and other regulators will be required to satisfy evidence requirements.
*GBS vaccine policy consideration gaps include:* Co-administration of vaccines in pregnancy (reactogenicity and immunogenicity) Persistence of GBS vaccine antibodies: would future pregnancies require repeat vaccination? The optimal timing of a GBS vaccine in pregnancy, considering the issue of prematurity Any potential for a GBS vaccine in pregnancy to affect infant primary immunisation responses (as has been seen with e.g. dTaP)?	Even if not a pre-licensure, regulatory requirement, studies to answer these questions will be useful from a policy perspective. Recommendations include: Results of a phase II vaccine candidate study, including co-administration with dTap, are expected to be available before UK policy is implemented. However, further co-administration data will be required to include the other vaccines recommended in pregnancy in the UK (COVID-19, pertussis and influenza vaccines). Timing of vaccination in pregnancy is also being looked at in ongoing studies but may need further policy revision post-implementation, with real-world evidence. Any effect of the GBS vaccine on infant immune responses is important and will need to be studied post-licensure. In addition, data on duration of protection is needed. For data on protection against LOGBS in the infant, and in vaccinees, to inform vaccination policy for future pregnancies Most of these issues may be investigated via phase IV studies, some at national level and some by academic institutions. Future meetings will expand on phase IV plans.
*Cost-effectiveness:* The GBS antenatal vaccine cost-effectiveness model for the UK will benefit from: Updated infant iGBS surveillance data Data on GBS related stillbirths Data on GBS related prematurity. The costs associated with premature birth can be very high, even if not frequently associated with GBS.	*Identifying potential data sources:* The proposed enhanced surveillance of infant GBS BPSU study will contribute updated incidence data A proposed study of GBS related stillbirths via a collaboration between SGUL, University of Oxford, the national Mothers and Babies: Reducing Risk through Audits and Confidential Enquiries collaboration (MBRRACE-UK), GBSS and perinatal pathologists.

### Advocacy and Engagement with Stakeholders

***A GBS vaccine in pregnancy programme: the midwifery perspective – Vanessa Greening and Emma Eccleston***

A presentation given by Vanessa Greening and Emma Eccleston, Research Midwives at SGUL Vaccine Institute, outlined some of the current barriers midwives might face in supporting pregnant people around GBS and GBS vaccines. Midwives may not always discuss GBS with pregnant people due to lack of time, staffing resource and confidence. Although very aware of the burden GBS places on maternity and paediatric services, midwives may lack specific knowledge of GBS incidence, mortality and morbidity rates.

Effective counselling on vaccines in pregnancy can be challenging; people may be hesitant discussing vaccines and when midwives lack confidence due to inadequate depth of training, their concerns may not be addressed. A study in 2013 asked London midwives their views on vaccination in pregnancy via an anonymous, online survey. ^38^ Just 25% of 266 respondents felt adequately prepared for this role and 69% of respondents agreed with the policy of vaccination against influenza in pregnancy.

Interestingly, many midwives themselves may be vaccine hesitant. UKHSA data for the 2021/22 seasonal influenza vaccine offer to frontline healthcare workers (HCWs) showed 75% uptake for nurses at St George’s Hospital, ^39^ but only 40% uptake amongst midwives at the same trust (local, unpublished data). Similarly, the 2013 survey study found seasonal influenza vaccine uptake rates in midwives of 43%, with non-uptake reasons including doubts about necessity, safety and effectiveness. ^38^

***What do parents need to know now? by Group B Strep Support – Jane Plumb***

Jane Plumb, Chief Executive Officer and Founder of Group B Strep Support (GBSS) charity reported on an unbranded survey GBSS conducted in April 2021, partnering with Bounty, to determine what information families already had about GBS in pregnant people and newborns. This was circulated online; the majority of respondents self-reported as pregnant people or having a child younger than 2 years of age.

3500 responses were obtained, and the results were summarised. 87% of respondents had heard of GBS, compared with a previous GBSS survey via a pregnancy and birth magazine when only 10% of respondents had. 54% of respondents had heard about GBS through their own personal experiences or via friends and only 14% from their midwives, with others citing pregnancy books, leaflets, websites, and social media. This demonstrates how information on GBS may come from non-academic, non-medical sources.

Importantly, 73% of respondents wanted more information on GBS, responding that they had not had ‘enough’ or ‘any’ information on the disease. Since 2017, the Royal College of Obstetrics and Gynaecology’s (RCOG) recommendation has been that all pregnant people should be provided with information on GBS; evidently, this target is not being met and more work needs to be done to provide access to resources on this infectious disease.

Almost all (99%) respondents felt that pregnant people should be informed about GBS by their doctor or midwife during pregnancy, 93% thought the UK should introduce a national screening programme for GBS in pregnancy (like other developed countries), selecting ‘extremely’ or ‘very important’ for screening policies and practices to be put in place. Finally, 90% of respondents answered they would be ‘very’ or ‘fairly’ likely to accept a GBS vaccine during pregnancy.

More recently, GBSS and charity partners conducted a repeat survey, advertised to those of child-bearing age; 321 responses were received at the time of this presentation in 2023 and data are both in progress and unpublished. When asked how important they felt different aspects of a GBS vaccine were, key concerns from respondents were protection of the baby against GBS infection and any side effects for the baby.

***What will GBS3 tell us and how do we use these results when thinking about GBS vaccines – Kate Walker***

Kate Walker, Professor of Obstetrics at Nottingham and Co-Chief Investigator of the GBS3 trial, gave an overview of the trials current progress. The GBS3 trial is a two-arm, parallel, cluster randomised trial of GBS testing strategies with an economic and acceptability evaluation. The sample size comprises 320,000 participants. Sites are randomised to one of two arms: routine testing or a risk-factor based strategy (usual care), with the routine testing sites further sub-randomised to either testing via intrapartum rapid PCR tests for GBS or via antenatal enriched culture medium (ECM) testing at 35-37 weeks gestation. Data is collected for 12 months with no individual data collected, except for a subset of 8000 participants. Aside from verbal consent for any swab taken, there is no written informed consent for the trial. The primary outcome is all-cause early neonatal sepsis. Data collection will continue until end of March 2024 with results expected Summer 2025.

The GBS3 trial results will remain relevant even with a GBS vaccine available as there will always be a requirement for GBS testing, for example for those who are unable to receive or access a vaccine. It was noted that during the COVID-19 pandemic, vaccine uptake in pregnancy was around 60% with just 30% uptake amongst Black pregnant people and 38% amongst the most deprived groups.

***What have we learnt from vaccination in pregnancy with COVID-19 vaccines? – Asma Khalil***

Asma Khalil, Professor of Fetal Maternal Medicine at SGUL presented on lessons learned from rapid rollout of a new vaccine in pregnancy during the COVID-19 pandemic. Approval of the first COVID-19 vaccine by the MHRA was in December 2020 but the JCVI did not recommend offering a COVID-19 vaccine in pregnancy until April 2021. Initial uptake was low, with figures at 2.8%. RCOG issued strong recommendations to offer vaccination in pregnancy in July 2021 followed by an intense campaign by RCOG and the government. Uptake improved following this campaign rising to 60% by January 2022.

- The RCOG campaigned for vaccinations in pregnancy using some of the below methods:
- Writing to the prime minister in November 2020 calling for more research trials in pregnancy
- Working closely with the vaccine taskforce
- Forming a vaccine subgroup to provide pregnant people and healthcare practitioners with advice on counselling, vaccine delivery and data collection.
- Producing information leaflets and decision aids, jointly with the Royal College of Medicine to help eligible pregnant people make a decision regarding vaccination.
- Social media has significant impact on public perception - both RCOG and the government used social media to promote facts and address priority groups with risk factors for poor uptake.

Looking at COVID-19 vaccine uptake and hesitancy in pregnancy multiple studies made similar findings. An SGUL study published in July 2021 ^28^ showed that approximately one in three pregnant people at St George’s hospital were accepting a COVID-19 vaccine in pregnancy. This SGUL study also examined the determinants of vaccine uptake. Multivariate regression analysis showed that there were three independent predictors of vaccine uptake: African-Caribbean ethnicity with an odds ratio (OR) of 0.27 (p value=0.044); those in the fifth (lowest) Indices of Multiple Deprivation (IMD) quintile had an OR 0.1 (p=0.003); and those with pre-gestational diabetes were 10.5 times more likely to get the vaccine (p=0.014). The ethnic group and socioeconomic disparities were demonstrated again in surveillance data from both England ^29^ and Scotland. ^40^ The role of healthcare professionals is an important determinant of vaccine uptake, particularly the advice given by midwives and obstetricians.

One of the key issues in vaccine implementation was addressing any safety issues quickly for pregnant individuals, particularly when a side effect becomes associated with a vaccine. From clinical practice experience, safety of the COVID-19 vaccine was the predominant concern to pregnant people, rather than efficacy.

Despite the RCOG’s efforts calling for pregnant people to be included in randomised-controlled trials (RCTs), it proved to be extremely difficult given that large numbers were needed, safety data was not yet available and sponsors were not always willing to recruit pregnant participants. There have been three RCTs examining the COVID-19 vaccine in pregnancy, none of which have reported data yet. An approach in future that doesn’t insist upon RCTs for vaccination in pregnancy is vital for timely rollout of antenatal vaccines.

### Advocacy and Engagement with Stakeholders: Knowledge Gaps Identified and Next Steps

Group discussion identified knowledge gaps in advocacy and education for a GBS vaccine programme in the UK. Table 3 details these knowledge gaps as well as next steps to begin to address these.

**Table 3.** Advocacy and engagement with key stakeholders: knowledge gaps identified and next steps.

Knowledge gaps identified	Next steps
*Education in pregnancy:* Midwives should feel empowered to inform people on both GBS and other licensed vaccines in pregnancy. Confidence to tackle difficult conversations in pregnancy, particularly around anti-vaccination or vaccine hesitancy issues, could be improved. Other healthcare professionals (HCPs) also lack sufficient time in clinical settings to counsel in detail on individual vaccines in pregnancy.	*The group proposed some education and training resources:* GBSS are developing a free, easily accessible GBS e-learning module for midwives and other HCPs. Additional modules could be added in future to address specific GBS vaccine education. Other education tool ideas included short videos, a short fact-sheet with frequently asked questions, prompts for midwives to discuss GBS and vaccines in pregnancy, multi-language resources, and midwife GBS Vaccine Champion(s).
*Possible vaccine hesitancy in midwives:* Given the lower uptake of influenza and also COVID-19 vaccines in midwives compared with some other staff groups, there is a need to understand and address any hesitancy around vaccine or research information in this vital clinical group.	A further study could examine potential vaccine hesitancy in midwives in more depth. Education resources, as listed above, could also specifically address this.
*Further engagement:* Engaging other stakeholders is essential to ensure equitable and rapid vaccine implementation.	Work with the Department of Health and Social Care (DHSC) to ensure expert stakeholders are able to assist with planning and preparation for effective and accessible vaccine implementation. Engagement with professional bodies such as RCOG and the Royal College of Midwives (RCM) to promote vaccine education to midwives and other HCPs. The RCOG ‘Green Top Guidelines’ on GBS will be updated in the coming years and information resources for families will be developed alongside these in collaboration with GBSS. Engagement with charities such as GBSS, sepsis and meningitis charities, as well as other pregnancy and baby charities and relevant patient groups to ensure cohesive vaccine messaging. Advocacy with and for groups at risk of lower vaccine uptake to promote levelling up of vaccine access.

### Considerations for GBS vaccine Phase IV Studies in the UK

Post-licensure vaccine effectiveness assessment, based on observational studies and the use of real-world evidence, requires high-quality disease surveillance systems to be in place with linkage to pregnancy vaccination systems. There are different ways in which these studies might be done and certain vaccine implementation strategies could allow for concurrent effectiveness data collection, including cluster or stepped wedge randomisation design to evaluate implementation. Endpoints of effectiveness and vaccine safety can be studied in various ways including case-control or passive cohort studies. Clinical effectiveness will need to be evaluated, with iGBS <90 days of age, both EOD and LOD, including bacteraemia and meningitis, as the endpoints. The implementation of the vaccine may lead to the discovery of other endpoints that GBS is contributing to, using a vaccine probe approach. This could yield a wide range of potential information regarding the role of GBS in invasive maternal disease and other maternal infections such as urinary tract infection or post-partum sepsis, as well as other neonatal infections (arthritis/cellulitis/culture negative sepsis), all-cause neonatal infections, stillbirths, prematurity and low birthweight, maternal and neonatal GBS colonisation, maternal and infant antibiotic use, serotype or strain replacement.

### UKHSA Overview of Potential Designs for a GBS Vaccine Phase IV Study

Nick Andrews first outlined the role and responsibilities of UKHSA when introducing a new vaccine in the UK. UKHSA have a legal mandate to undertake surveillance without patient consent with responsibility for evaluating several areas after vaccine introduction:

- Disease incidence (pre- to post-vaccine introduction)
- Epidemiological impact (for example, age shift, season shift, strain changes)
- Vaccine effectiveness and safety (with MHRA)
- Vaccine coverage and inequalities
- Cost-effectiveness.

This is achieved using a variety of data sources including laboratory data from SGSS, vaccine registers, GP databases, hospital and mortality data, enhanced surveillance via questionnaires to GPs may be used (especially effective for rare diseases), and case note review. UKHSA also considers the vaccine schedule and the need for additional immunogenicity evaluation in trials, for example with the National Immunisation Schedule Evaluation Consortium (NISEC).

GBS vaccine phase IV studies may be able to take similar approaches to previous pathogens. Some examples of previous phase IV studies carried out in the UK post-vaccine introduction are outlined in Table 4.

**Table 4.** An overview of possible GBS vaccine phase IV study designs with previous examples.

Study design	Previous examples	Details
Case-coverage (screening) approach	Meningococcal B, ^41^^,^^42^ C, ACWY Vaccine effectiveness. Antenatal pertussis vaccine effectiveness and safety ^43^	Obtain the vaccination status of cases and match to population uptake. Coverage data from a subset of Child Health Information Systems. Case data from laboratory reports with vaccination status found by contacting GPs. Coverage data from a subset of the population using the Clinical Practice Research Datalink (CPRD) from GP surgeries. Case data was from laboratory reports, vaccination status and other clinical details obtained by contacting GPs.
Test-negative case-control study	COVID-19 vaccine in pregnancy effectiveness ^44^	Data sources were infant cases and controls from the national ‘pillar 1 and 2’ testing. Pillar 1 was SARS-CoV-2 PCR testing performed within UKHSA laboratories or NHS hospital services and pillar 2 was wider community testing. Mothers identified using linkage via the Maternal Services Dataset (MSDS). Vaccination data was from the National Immunisation Management System (NIMS)
Nested case-control study (within retrospective cohort study)	COVID-19 vaccine in pregnancy safety ^45^	Mothers identified using linkage via the Maternal Services Dataset (MSDS). Vaccination data was from NIMS Safety data was from Hospital Episodes Statistics (HES), Secondary Uses Services (SUS) and MSDS datasets. COVID-19 testing data from pillar 1 and 2 services as above.
*Indirect Cohort (Broome Method)*	*Pneumococcal vaccine effectiveness* ^46^	*This approach would likely NOT be relevant for GBS: involves looking at non-vaccine serotype cases, which would likely be insufficient in number to power a study.*

It was noted that the approach used for COVID-19 vaccine in pregnancy may have most relevance to GBS. Linkage to NIMS was very effective for these studies. As a national register, NIMS removed the need to try to access GP vaccination data, which is time-consuming. Replicating this approach will depend on whether GBS vaccines will be recorded in NIMS or via GP records, and, if into GP records first, whether a national extraction from these records can be done that allows linkage. This may be dependent on the vaccine delivery setting, for example vaccination via GP versus maternity services.

***Potential phase IV study designs and relevant data sources for GBS include:***

1. Vaccine effectiveness estimate:
  With just laboratory surveillance data, UKHSA could assess the impact pre- and post-introduction on infants with GBS, mothers with GBS, epidemiology and strains.
2. A nationwide cohort study:
  With access to a vaccine register (such as NIMS, which provides denominator data), using vaccination records and linking infant cases to mothers using a programme such as MSDS, UKHSA could set up a cohort of the entire country. Identifying cases and matching them to the denominator. A cohort using vaccine register linkage is particularly good for multiple endpoints.
3. A cohort for safety assessment:
  Using MSDS linked to a vaccine register and HES or within a GP database (e.g., CPRD).
4. A nested, matched, case-control study:
  If additional information on confounders is needed, with access to a vaccine register, linkage to MSDS and to SGSS laboratory reports (including serotype data) a case-control study could be set up with a suitable laboratory control. Although this is likely not necessary.

Without access to a vaccine register, the case-coverage approach or a small cohort study could be used, both necessitating contacting GPs for vaccination status, which is less efficient. Infant/mother linkage data would still be required. Other endpoints could be evaluated using these proposed study designs, for example stillbirth, within a vaccine probe study.

Considering the possibility of effectiveness data collection alongside vaccine implementation, for example stepped wedge or cluster randomisation implementation design, it was noted that these methodologies are infrequently used due to more complex feasibility and ethical considerations. Previous antenatal vaccines introduced into the UK, for example the pertussis vaccine, have had highly protective effects, making these types of observational studies more useful. For a vaccine with lower effectiveness, these studies become challenging. This would also be true of a vaccine probe study; better results are yielded at higher effectiveness levels. At lower effectiveness, the benefits of cluster randomised or stepped wedge implementation design might be more apparent. It was noted that a GBS vaccine is anticipated to be highly effective against the serotypes and strains included but with monitoring for serotype or strain replacement.

Further discussion and development of plans for phase IV studies is needed. Many of these will be primarily carried out at national level by UKHSA within their remit for evaluation of vaccine implementation. In addition to these large-scale effectiveness and safety studies, there are other research questions better suited to smaller scale studies than by public health bodies. SGUL is already involved in several areas of work to progress GBS vaccine implementation in the UK, both at pre-licensure and post-licensure stage. This work is in close collaboration with charity partners, such as GBSS, regulators including the MHRA, government departments and industry partners. This multi-agency approach is vital for an effective and accessible vaccine programme. Some of these areas of work, current and planned, are outlined below:

*Pre-vaccine implementation:*

- Defining a COP for GBS and engagement with regulators.
  ○ Several studies are co-ordinated by SGUL to work towards defining a COP, applicable to the UK
- National level enhanced GBS surveillance data ahead of vaccine introduction
  ○ The proposed BPSU study of infant GBS across the UK and Ireland, led by SGUL, will provide important data updates, supported by GBSS and in collaboration with public health agencies from across the UK and Ireland.
- Advocacy and engagement with patient and public groups, including charities such as GBSS, meningitis and sepsis charities such as the Meningitis Research Foundation (MRF), Meningitis Now and the UK Sepsis Trust, as well as with pregnancy and baby charities.
  ○ GBSS, for example, continues to work towards better education of healthcare professionals and the public to raise awareness of disease risks and improve access to information
  ○ This may increase vaccine acceptability and so eventual vaccine uptake
  ○ Engagement is needed specifically with groups at risk of poor vaccine uptake and addressing barriers to vaccine access
- Engagement with professional bodies such as RCOG, RCM and the Royal College of General Practitioners (RCGP) all of whom can have far reaching influence on their clinical staff members.
- Engagement with government departments such as the Department for Health and Social Care (DHSC)
  ○ To ensure a GBS vaccine is prioritised for implementation and working towards equitable vaccine coverage

*Post-vaccine implementation:*

- Continued engagement with charities, DHSC and others working with patient groups to advocate strongly for rapid implementation, uptake of a vaccine programme and maintaining a focus on equitable access given the knowledge of higher incidence rates of iGBS disease in groups at risk of low vaccine uptake.
- Evidence generation – across multiple research questions:
  ○ Vaccine access: understanding disparities in under-vaccinated groups, attitudinal studies
  ○ Vaccine schedules in pregnancy: co-administration of antenatal vaccines, timing of administration, effects on infant primary immunisation responses
  ○ Vaccine failure: risk factors, characterisation
  ○ Special groups: vaccine safety in immunocompromise, those with co-morbidities, lactating people
  ○ Effects on use of IAP pre/post vaccine introduction

## Conclusions to the Meeting

This meeting was the first, round table discussion in the UK on GBS vaccines and has opened conversations between key stakeholders for implementation. A concerted and co-ordinated effort across multiple workstreams and organisations will be required to introduce a GBS vaccine that is accepted by, and accessible to, everyone and especially those with the greatest need in the UK.

Further round table meetings are planned to gather stakeholders together again and ensure continued progress towards this goal.

To summarise the key areas identified at this meeting where action is needed:

*Data and evidence gaps*

Additional data ahead of vaccine implementation would help direct and strengthen arguments for vaccine policy, for example updated enhanced surveillance data, data on GBS related stillbirths.

*Vaccine licensure*

Work is ongoing to progress vaccine candidates towards licensure including planned phase III trials and seroepidemiological studies for COP development in liaison with regulators.

*Phase IV studies*

Plans for phase IV studies need further detailed discussion and planning to progress. More creative approaches for implementation alongside effectiveness data collection will be considered. National level phase IV studies by UKHSA will be complemented by smaller-scale studies by SGUL and others to hone in on specific questions relating to specific groups.

*Education and advocacy opportunities*

Improved access to education for midwives on GBS and GBS vaccines as well as other HCP groups will likely have a positive impact on vaccine acceptability to both HCPs and the public. Advocating for rapid implementation of a GBS vaccine will be important, alongside work to maximise uptake and level-up access to a vaccine in the UK.

### List of Meeting Attendees

**Chairs:**

Kirsty Le Doare – Professor of Vaccinology and Immunology, St George’s University, Centre for Neonatal and Paediatric Infection

Paul Heath – Professor of Paediatric Infectious Diseases, St George’s University, Centre for Neonatal and Paediatric Infection

**Attendees (A-Z):**

Arlene Reynolds – Senior Professional Adviser in Public Health in Scottish Government, Previously Public Health Scotland

Asma Khalil – Professor of Fetal Maternal Medicine at St George’s University, London

Caroline Trotter – Epidemiologist, Cambridge and Imperial College Universities

Cecilia Hultin – Senior Research Nurse St George’s University Vaccine Institute

Cheryl Battersby – Consultant Neonatologist at Chelsea and Westminster Hospital, NIHR Clinician Scientist

Claire Sharkey – Clinical Research Fellow at St George’s University Vaccine Institute

Claire Wright – Head of Evidence and Policy, Meningitis Research Foundation

Colin Brown – Director of Clinical Emerging Infection at UKHSA

Debbie King – Research Lead, Vaccines at Wellcome Trust

Elaine Devine – Director of Communications and Advocacy, Meningitis Research Foundation

Elizabeth Hayter – Clinical Trials Co-Ordinator St George’s University Vaccine Institute

Emma Eccleston – Research Midwife at St George’s University Vaccine Institute

Eva Galiza – Senior Clinical Research Fellow, St George’s University, Centre for Neonatal and Paediatric Infection

Helen Campbell – Clinical Scientist, Immunisations Team at UKHSA

Jane Plumb – Chief Executive of Group B Strep Support (GBSS)

Juliana Coelho – National Infection Service: Respiratory and Vaccine Preventable Bacteria Reference Unit (NIS:RVPBRU), UKHSA

Kate Walker – Professor of Obstetrics at Nottingham University, Co-Chief Investigator of GBS3 Trial

Konstantinos Karampatsas – Clinical Research Fellow, St George’s University, Centre for Neonatal and Paediatric Infection

Laura McDonald – Health Care Scientist, Immunisation team, Public Health Scotland

Lauren Wallis – Communications/Engagement Officer at St George’s University, Centre for Neonatal and Paediatric Infection

Lynne Rush – Public Health trainee, Immunisation team at Public Health Scotland

Mair Powell – Senior Clinical Assessor at the Healthcare Products Regulatory Authority, Ireland,

Merryn Voysey – Associate Professor of Statistics in Vaccinology, Oxford Vaccine Group

Michelle Falconer – Nurse Consultant, Vaccine and Immunisations, Public Health Scotland

Natasha Thorn – Clinical Research Fellow at St George’s University, Centre for Neonatal and Paediatric Infection

Nick Andrews – Statistician at UKHSA

Oliver Plumb – Advocacy and Information Manager, Group B Strep Support (GBSS)

Phil Steer – Chair of Medical Advisory Committee, Group B Strep Support (GBSS)

Rebecca L Guy – Epidemiologist at UKHSA

Sam Knight – Helpline & Information Officer at Group B Strep Support (GBSS)

Shamez Ladhani – Paediatrician and UKHSA Epidemiologist

Theresa Lamagni – Epidemiologist, Healthcare Acquired Infections and Antimicrobial Resistance, UKHSA

Vanessa Greening - Research Midwife, St George’s University Vaccine Institute

Xinxue Liu – Statistician, Oxford Vaccine Group

## Ethical considerations

No relevant ethical considerations for this article and no ethics committee approval sought.

## Consent

Written consent via email was gained from all presenters to publish their presentation content.

## Data Availability

The data for this article consists of bibliographic references, which are included in the References section.
