# Supplementary material for: GBS vaccines in the UK: a round table discussion
Source: F1000Res. 2024 May 21;13:519. [Version 1] doi: 10.12688/f1000research.147555.1 (PMC11350325; doi:10.12688/f1000research.147555.1)
Supplement: Peer review history

# Reviewer report: Reviewer response for version 1

**DOI:** 10.5256/f1000research.161761.r315651

**Published:** 2024-09-26

**Authors:** Rosana Rocha Barros

**Affiliations:** 1 Federal Fluminense University, Niterói, State of Rio de Janeiro, Brazil

Revision of the manuscript

 GBS vaccines in the UK: a round table discussion

 The manuscript is a comprehensive report of the round table held at St George University of London, that discussed the state of the art of GBS vaccines and planned phase IV trials. The manuscript brings the talks of different specialists, covering various issues regarding GBS vaccine background, vaccine implementation, and the follow-up after the beginning of vaccination. Overall, the text is very well-written and I have only an observation, as follows.

 Page 3

 2 ^nd^ paragraph.

 “IAP is not always deliverable, results in high antibiotic exposure...” This sentence seems a bit unclear. I suggest that the authors improve it.

Is the work clearly and accurately presented and does it cite the current literature?

Yes

If applicable, is the statistical analysis and its interpretation appropriate?

Not applicable

Are all the source data underlying the results available to ensure full reproducibility?

No source data required

Is the study design appropriate and is the work technically sound?

Yes

Are the conclusions drawn adequately supported by the results?

Yes

Are sufficient details of methods and analysis provided to allow replication by others?

Yes

Reviewer Expertise:

Microbiology

I confirm that I have read this submission and believe that I have an appropriate level of expertise to confirm that it is of an acceptable scientific standard.

# Reviewer report: Reviewer response for version 1

**DOI:** 10.5256/f1000research.161761.r315650

**Published:** 2024-09-10

**Authors:** Hannah R Frost

**Affiliations:** 1 Murdoch Children's Research Institute, Parkville, Victoria, Australia

Thank you for the invitation to review the manuscript "GBS vaccines in the UK: a round table discussion" by Thorn et al., it was an interesting and informative read. The article provides a concise and well-rounded update to the current status of GBS Vaccines, including an appropriate focus on knowledge gaps and barriers to success with useful recommendations for how to address them. It is particularly interesting to have an update on important ongoing or planned trials, which is not normally available in the literature until >1 year after the completion of the trial. I appreciate the focus on forward planning around vaccine uptake and phase IV trials, and keeping in mind lessons from COVID-19 and other vaccines given in pregnancy. 

 I have a few minor comments which may improve readability of the manuscript.

 1) There is some repetition of points throughout, likely due to the nature of the manuscript as proceedings of a meeting. The authors could clean up the narrative, for example on page four, two subsequent paragraphs have the same conclusion regarding the need for improved surveillance. 

 2) Different acronyms are used to refer to the same thing (e.g. EOGBS, EOD and EO disease are all used in the first page) and some acronyms are never expanded (e.g. UR when discussing case estimates). 

 3) It would be good to have references and links provided for the burden of disease data used, acknowledging that some data is as yet unpublished.

Is the work clearly and accurately presented and does it cite the current literature?

Yes

If applicable, is the statistical analysis and its interpretation appropriate?

Yes

Are all the source data underlying the results available to ensure full reproducibility?

Partly

Is the study design appropriate and is the work technically sound?

Yes

Are the conclusions drawn adequately supported by the results?

Yes

Are sufficient details of methods and analysis provided to allow replication by others?

Yes

Reviewer Expertise:

I work in infection and immunity, with specific focus on accelerating vaccine development for Group A Streptococcus.

I confirm that I have read this submission and believe that I have an appropriate level of expertise to confirm that it is of an acceptable scientific standard.

# Reviewer report: Reviewer response for version 1

**DOI:** 10.5256/f1000research.161761.r315656

**Published:** 2024-08-27

**Authors:** Lisa Hanson

**Affiliations:** 1 Marquette University, Milwaukee, USA

*Very well written article. Statistics and references are up to date and appropriate. Tables are very effective.

 A few suggestions for clarity.

 *"more crowded pregnancy vaccine space" is unclear.

 *In the GBS3 trial description, more clarity is needed as to why participants in the routine testing arms receive either rapid PCR IP (versus 35-37 weeks). A reference here about the sensitivity and utiliy of rapid IP testing is needed-as this is not a usual strategy in culture-based EOGBS prevention approach recommended by the CDC and now ACOG (2019).

 *Table 3. The points about midwives having hesitancy to offer vaccines was interesting, as this is not the case in the USA.

 *Table 4 is redundant of the text on Potential Phase IV study designs.

Is the work clearly and accurately presented and does it cite the current literature?

Yes

If applicable, is the statistical analysis and its interpretation appropriate?

Not applicable

Are all the source data underlying the results available to ensure full reproducibility?

No source data required

Is the study design appropriate and is the work technically sound?

Yes

Are the conclusions drawn adequately supported by the results?

Yes

Are sufficient details of methods and analysis provided to allow replication by others?

Yes

Reviewer Expertise:

Antenatal probiotics to reduce GBS colonization.

I confirm that I have read this submission and believe that I have an appropriate level of expertise to confirm that it is of an acceptable scientific standard.

# Reviewer report: Reviewer response for version 1

**DOI:** 10.5256/f1000research.161761.r304243

**Published:** 2024-08-07

**Authors:** Jennifer Gaddy

**Affiliations:** 1 Vanderbilt University, Nashville, Tennessee, USA

The manuscript by Natasha Thorn and colleagues entitled, “GBS vaccines in the UK: a round table discussion” presents a compelling discussion of the status of a protective vaccine against Group B Streptococcus, an important perinatal pathogen.  This manuscript is full of important information about disease risk from GBS infection and gaps in current treatment and prevention strategies.  There are many positive aspects about this manuscript that I would like to highlight. 

 First, the authors are extremely deliberate in their use of language, specifically referring to “pregnant patients” and “pregnant people”.  This is a subtle but important aspect of discussing these populations without introducing highly gendered language. Excellent work.

 The inclusion of stakeholders in the community such as Midwives was also a strength as these providers have the capacity to meet individuals who may be unaware of GBS risk and/or vaccine hesitant.  Buy-in from these groups will help with deployment in the future.

 Comparing/contrasting efficacy of other vaccination programmes deployed in pregnant patients was also a strength of this manuscript.

 I have a few comments to improve the quality of the manuscript.

 1.  The authors mention AMR very briefly in the second paragraph of the Introduction.  It would be helpful to expand this section to acknowledge that the standard first line therapeutic choice for GBS is penicillin, but up to 10% of populations report penicillin hypersensitivity. Second line choice is often erythromycin or clindamycin and emerging clinical strains are exhibiting high resistance to these drugs (about 40% of strains are resistant). 

 2.  First line of the Introduction.  The authors refer to Group B streptococcus and italicize the word “streptococcus” but leave it lowercase.  If the authors are referring to the genus, this word should be capitalized and italicized. If they are referring to general morphology and arrangement of bacteria it can be lowercase but should not be italicized.  Most common references to GBS use the former (genus nomenclature).

Is the work clearly and accurately presented and does it cite the current literature?

Yes

If applicable, is the statistical analysis and its interpretation appropriate?

Not applicable

Are all the source data underlying the results available to ensure full reproducibility?

No source data required

Is the study design appropriate and is the work technically sound?

Yes

Are the conclusions drawn adequately supported by the results?

Yes

Are sufficient details of methods and analysis provided to allow replication by others?

Yes

Reviewer Expertise:

I work in reproductive infection and immunology, specifically in the area of Group B Streptococcus pathogenesis and host responses to infection.

I confirm that I have read this submission and believe that I have an appropriate level of expertise to confirm that it is of an acceptable scientific standard.

# Response

**Published:** 2024-08-12

**Authors:** Natasha Thorn

**Affiliations:** Paediatric Infection, St George's University, London, London, UK

Thank you for this helpful and insightful peer-review. We appreciate your time and positive comments on our manuscript. 

 We will certainly address your two points of suggestion. Thank you for highlighting the typographical error in point 2. 

 Regarding discussion of AMR in the article, in addition to paragraph 5 of the "Epidemiology and surveillance" section, we will include some additional context in the manuscript on penicillin hypersensitivity to further highlight the impact of clindamycin resistance. 

 We are awaiting a second peer review and will then make the above changes to the manuscript.
